# Author Correction: Repression of phagocytosis by human CD33 is not conserved with mouse CD33

**DOI:** 10.1038/s42003-020-0769-8

**Published:** 2020-01-16

**Authors:** Abhishek Bhattacherjee, Emily Rodrigues, Jaesoo Jung, Matthew Luzentales-Simpson, Jhon R. Enterina, Danny Galleguillos, Chris D. St. Laurent, Maryam Nakhaei-Nejad, Felix F. Fuchsberger, Laura Streith, Qian Wang, Norihito Kawasaki, Shiteng Duan, Arjun Bains, James C. Paulson, Christoph Rademacher, Fabrizio Giuliani, Simonetta Sipione, Matthew S. Macauley

**Affiliations:** 1 Department of Chemistry, University of Alberta, Alberta, Canada; 2 Department of Medical Microbiology and Immunology, University of Alberta, Alberta, Canada; 3 Department of Pharmacology, University of Alberta, Alberta, Canada; 4 Department of Medicine, University of Alberta, Alberta, Canada; 5 Department of Biomolecular Systems, Max Planck Institute of Colloids and Interfaces, Potsdam, Germany; 6 Department of Molecular Medicine, The Scripps Research Institute, La Jolla, CA 92037 USA

**Keywords:** Neuroimmunology, Microglia

Correction to: *Communications Biology* 10.1038/s42003-019-0698-6, published online 03 December 2019.

In the original published version of the article, funding information for co-author James C. Paulson was inadvertently omitted from the acknowledgements. The error has been corrected in the HTML and PDF versions.

