# Supplementary material for: Repression of phagocytosis by human CD33 is not conserved with mouse CD33
Source: Commun Biol. 2019 Dec 3;2:450. doi: 10.1038/s42003-019-0698-6 (PMC6890642; doi:10.1038/s42003-019-0698-6)
Supplement: Supplementary file 1 — Supplementary Information [file 42003_2019_698_MOESM1_ESM.pdf]

Supplementary Data

Supplementary Figure 1

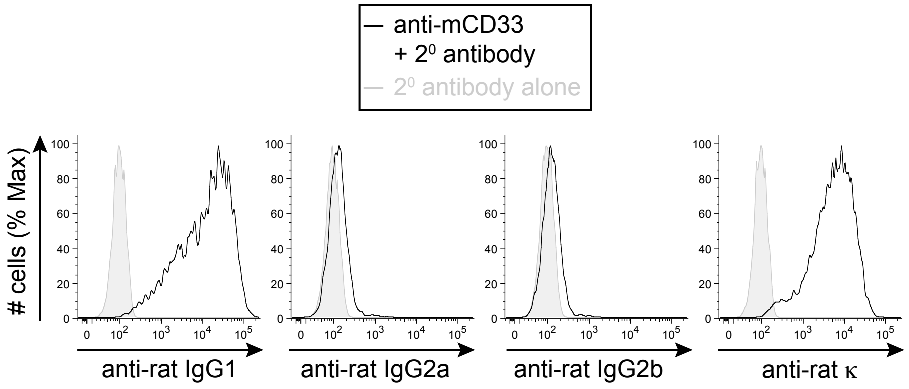

Supplementary Figure 1: Characterization of anti-mouse CD33 monoclonal antibody. CHO cells expressing the extracellular portion of mCD33 were stained with supernatant from the clone 9A11 antibody was followed up with the indicated secondary PE-conjugated antibodies and analyzed by flow cytometry.

Supplementary Figure 2

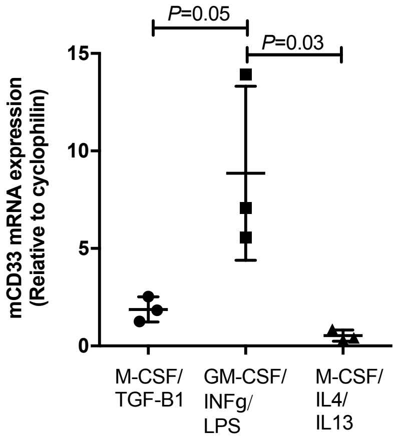

Supplementary Figure 2: Differential expression of mCD33 mRNA in polarized neonatal expanded microglia. Neonatal microglia were isolated and expanded from the brain of neonatal WT mice and differentiated with the indicated conditions. mRNA expression of mCD33 and cyclophilin were quantified by qPCR and values are standardized to the cyclophilin. Error bars represent +/- standard deviation of three replicates. Statistical significance calculated based on an unpaired Student's T-test.

Supplementary Figure 3

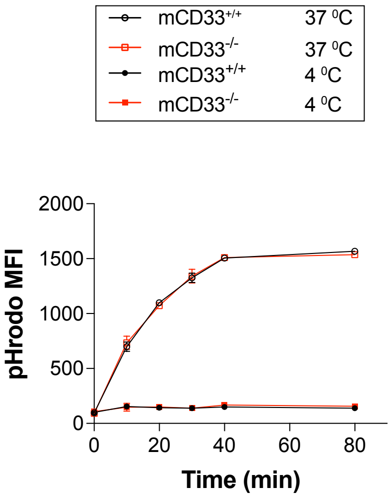

Supplementary Figure 3: Time- and temperature-dependent uptake of pHrodo-myelin in RAW264.7 cells. WT (black) and mCD33$^{-/-}$ (red) were incubated with pHrodo-myelin for different amounts of time at 4 ^{0}C (closed symbols) and 37 ^{0}C (open symbols) and analyzed by flow cytometry for the mean fluorescence intensity (MFI) of the pHrodo fluorophore. Error bars represent +/- standard deviation of three replicates.

Supplementary Figure 4

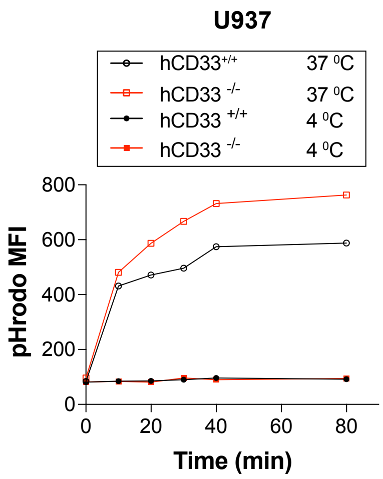

Supplementary Figure 4: Time- and temperature-dependent uptake of pHrodo-myelin in U937 cells. WT (black) and hCD33$^{-/-}$ (red) were incubated with pHrodo-myelin for different amounts of time at 4 ^{0}C (closed symbols) and 37 ^{0}C (open symbols) and analyzed by flow cytometry for the mean fluorescence intensity (MFI) of the pHrodo fluorophore. Error bars represent +/- standard deviation of three replicates.

Supplementary Figure 5

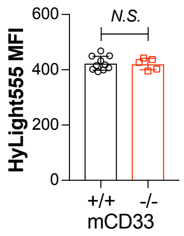

Supplementary Figure 5: Cellular uptake Hylight555-labeled aggregated Aβ_{1-42} in undifferentiated neonatal microglia. Neonatal microglia from the brain of WT (black) and mCD33$^{-/-}$ (red) mice were incubated with aggregated Aβ_{1-42} for 30 minutes and analyzed by flow cytometry. Each data point represents microglia from different mice. Values represent the mean fluorescent intensity (MFI) of cells incubated with the cargo subtracted from the MFI of unstained cells, and error bars represent the standard deviation. Statistical significance was calculated using an unpaired Student's T-test; *N.S.* represented no statistical significance.

Supplementary Figure 6

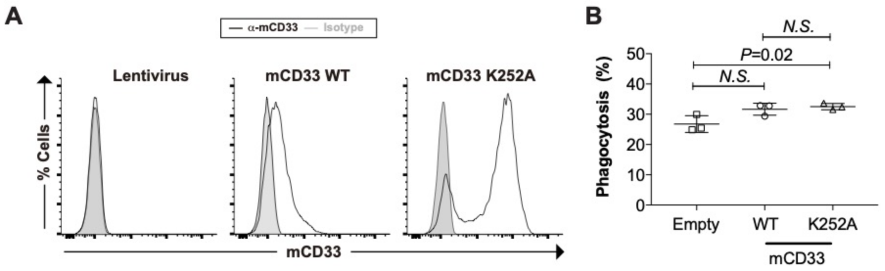

Supplementary Figure 6: Ectopic expression of mCD33 in U937 cells does not repress phagocytosis. hCD33$^{-/-}$ U937 cells were transduce with lentivirus expressing WT mCD33, K252A mCD33, or a control lentivirus. Three independent transductions were carried out to control for clonal variability. **(A)** Expression of mCD33 in one of the representative replicates of the three cell types by flow cytometry. **(B)** Uptake of polystyrene beads in the three replicates of the three cell types was determined by flow cytometry. Values represent % of cells taking up the beads (Pacific Blue positive); error bars represent the standard deviation. Statistical significance was calculated using an unpaired Student's T-test; *N.S.* represented no statistical significance.

Supplementary Figure 7

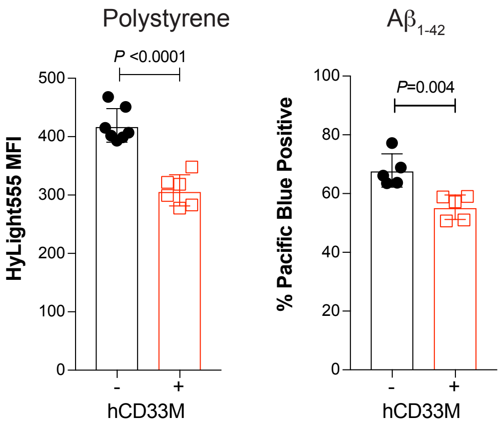

Supplementary Figure 7: Cargo uptake is suppressed in neonatal microglia expressing hCD33. Neonatal microglia from the brain of WT ($Cx_3cr1^+hCD33^-$) or hCD33-Tg ($Cx_3cr1^+hCD33^+$) mice under non-polarizing conditions. Uptake of Hylight555-labeled aggregated $A\beta_{1\text{-}42}$ (*left panel*) and polystyrene beads (*right*) was analyzed in cells and analyzed by flow cytometry; each data point represents microglia isolated from a different mouse. For uptake of $A\beta_{1\text{-}42}$, Values represent the mean fluorescent intensity (MFI) of cells incubated with the cargo subtracted from the MFI of unstained cells, whereas for uptake of polystyrene values represent % of cells taking up the beads (Pacific Blue positive); error bars represent the standard deviation. Statistical significance was calculated using an unpaired Student's T-test; *N.S.* represents no statistical significance.
