# Supplementary material for: Repression of phagocytosis by human CD33 is not conserved with mouse CD33
Source: Commun Biol. 2019 Dec 3;2:450. doi: 10.1038/s42003-019-0698-6 (PMC6890642; doi:10.1038/s42003-019-0698-6)
Supplement: Supplementary file 2 — Reporting Summary [file 42003_2019_698_MOESM2_ESM.pdf]

nature research

Corresponding author(s): Matthew Macauley

Last updated by author(s): Oct 23, 2019

Reporting Summary

Nature Research wishes to improve the reproducibility of the work that we publish. This form provides structure for consistency and transparency in reporting. For further information on Nature Research policies, see Authors & Referees and the Editorial Policy Checklist.

Statistics

For all statistical analyses, confirm that the following items are present in the figure legend, table legend, main text, or Methods section.

n/a	Confirmed	
☐	☒	The exact sample size (n) for each experimental group/condition, given as a discrete number and unit of measurement
☐	☒	A statement on whether measurements were taken from distinct samples or whether the same sample was measured repeatedly
☐	☒	The statistical test(s) used AND whether they are one- or two-sided<br>*Only common tests should be described solely by name; describe more complex techniques in the Methods section.*
☐	☒	A description of all covariates tested
☐	☒	A description of any assumptions or corrections, such as tests of normality and adjustment for multiple comparisons
☒	☐	A full description of the statistical parameters including central tendency (e.g. means) or other basic estimates (e.g. regression coefficient) AND variation (e.g. standard deviation) or associated estimates of uncertainty (e.g. confidence intervals)
☐	☒	For null hypothesis testing, the test statistic (e.g. F, t, r) with confidence intervals, effect sizes, degrees of freedom and P value noted<br>*Give P values as exact values whenever suitable.*
☒	☐	For Bayesian analysis, information on the choice of priors and Markov chain Monte Carlo settings
☒	☐	For hierarchical and complex designs, identification of the appropriate level for tests and full reporting of outcomes
☒	☐	Estimates of effect sizes (e.g. Cohen's d, Pearson's r), indicating how they were calculated

Our web collection on statistics for biologists contains articles on many of the points above.

Software and code

Policy information about availability of computer code

Data collection	BD FACSDivaTM software, CellReporterXpress, Molecular Devices.
Data analysis	Graph pad prism 7, FlowJo LLC.

For manuscripts utilizing custom algorithms or software that are central to the research but not yet described in published literature, software must be made available to editors/reviewers. We strongly encourage code deposition in a community repository (e.g. GitHub). See the Nature Research guidelines for submitting code & software for further information.

Data

Policy information about availability of data

All manuscripts must include a data availability statement. This statement should provide the following information, where applicable:

- Accession codes, unique identifiers, or web links for publicly available datasets
- A list of figures that have associated raw data
- A description of any restrictions on data availability

All the figures presented in the manuscript is associated with Raw data which will be available upon request

Field-specific reporting

Please select the one below that is the best fit for your research. If you are not sure, read the appropriate sections before making your selection.

☒ Life sciences ☐ Behavioural & social sciences ☐ Ecological, evolutionary & environmental sciences

For a reference copy of the document with all sections, see nature.com/documents/nr-reporting-summary-flat.pdf

Life sciences study design

All studies must disclose on these points even when the disclosure is negative.

Sample size	For human and mouse cell lines sample size varies from 5-7 biological repetition. In animal experiments sample size varies from 5-11 for each group.
Data exclusions	N/A
Replication	3-5 analytical replication done for cell line studies and 5-11 replication done for animal studies.
Randomization	N/A
Blinding	N/A

Reporting for specific materials, systems and methods

We require information from authors about some types of materials, experimental systems and methods used in many studies. Here, indicate whether each material, system or method listed is relevant to your study. If you are not sure if a list item applies to your research, read the appropriate section before selecting a response.

Materials & experimental systems

n/a	Involved in the study	
☐	☒	Antibodies
☐	☒	Eukaryotic cell lines
☒	☐	Palaeontology
☐	☒	Animals and other organisms
☒	☐	Human research participants
☒	☐	Clinical data

Methods

n/a	Involved in the study	
☒	☐	ChIP-seq
☐	☒	Flow cytometry
☒	☐	MRI-based neuroimaging

Antibodies

Antibodies used	CD11c (AF488, clone N418, BioLegend), CD19 (BV785, clone 6D5, BioLegend), CD3 (BV650, clone 17A2, BioLegend), B220 (BUV395, clone 6B2, BD Horizon), NK1.1 (BV421, clone PK136, Biolegend), mCD33 (PE, clone 9A11, eBioscience), CX3CR1(AlexaFluor 647, clone SA011F11, BioLegend), Ly-6C (APC/Cy7, clone HK 1.4, BioLegend), Ly-6G (PerCP/Cy5.5, clone 1A8, BioLegend), F4/80 (BV711, clone T45-2342, BD Horizon), Cd11b (BV510, clone M1/70, BioLegend), MHC-II (BV605, clone M5/114,15.2, BioLegend), and Siglec-H (PE-Cy7, clone eBio440C, Invitrogen). For analysis of microglia, the following cocktail was used: F4/80 (BUV395, clone T45-2342, BD Horizon), LY-6G (BV605, clone 1A8, BioLegend), CD11b (APC/Cy7, clone M1/70, BioLegend), Cx3cr1 (PerCP/Cy5.5, clone SA011F11, BioLegend) and Ly-6C (BV711, clone HK 1.4, BioLegend).For mCD33-/- versus mCD33+/+ analysis, we additionally used the following antibodies: CD45.1 (AF488, clone A20, BioLegend) and CD45.2 (BV785, clone 104, Biolegend).
Validation	All the antibodies commercially purchased from these companies: link provided :1) https://www.biolegend.com/ ; 2) https://www.thermofisher.com/ca/en/home/life-science/antibodies/ebioscience.html; 3) https://www.thermofisher.com/ca/en/home/brands/invitrogen.html?gclid=EAIaIQobChMIwt3qwOPk4gIVDcRkCh2kdQZbEAAYASAAEgLi-PD_BwE&s_kwcid=AL!3652!3!177826480722!b!!g!!%2Binvitrogen&ef_id=EAIaIQobChMIwt3qwOPk4gIVDcRkCh2kdQZbEAAYASAAEgLi-PD_BwE:G:s&s_kwcid=AL!3652!3!177826480722!b!!g!!%2Binvitrogen&mkwid=s-dc_pcrid_177826480722_pkw_+invitrogen_pmt_b_slid__dimid=; 4) http://www.bdbiosciences.com/ca/applications/research/multicolor-flow/m/745795/horizon. Each of the antibody can be searched by their clone numbers in respective website.

Eukaryotic cell lines

Policy information about cell lines

Cell line source(s)	ATCC
Authentication	All the parental cell lines were purchased from ATCC and they are namely:THP-1, U937, and RAW264.7
Mycoplasma contamination	No Mycoplasma contamination detected (ABM Mycoplasma detection kit, Canada)
Commonly misidentified lines (See ICLAC register)	N/A

Animals and other organisms

Policy information about studies involving animals; ARRIVE guidelines recommended for reporting animal research

Laboratory animals	All mice were on a C57BL/6J genetic background. WT, CD45.1+/+, Cx3cr1Cre, mCD33-/- and hCD33Tg mice were obtained from The Jackson Laboratory.
Wild animals	N/A
Field-collected samples	N/A
Ethics oversight	All animals used were maintained in an access-controlled barrier facility under specific-pathogen-free conditions. Studies were performed in accordance with Public Health Service guidelines and approved by the Animal Subjects Committee of the University of Alberta and the IACUC of The Scripps Research Institute.

Note that full information on the approval of the study protocol must also be provided in the manuscript.

Flow Cytometry

Plots

Confirm that:

☒ The axis labels state the marker and fluorochrome used (e.g. CD4-FITC).

☒ The axis scales are clearly visible. Include numbers along axes only for bottom left plot of group (a 'group' is an analysis of identical markers).

☒ All plots are contour plots with outliers or pseudocolor plots.

☒ A numerical value for number of cells or percentage (with statistics) is provided.

Methodology

Sample preparation	Described in methodology section of the manuscript.
Instrument	BD LSRFortessa TM X-20
Software	BD FACSDivaTM software V8.0.1
Cell population abundance	2000-100000 depending on assay.
Gating strategy	Gating strategy descried in detail under methodology section of the manuscript. Gating strategy for each analysis demonstrated in respective figures.

☐ Tick this box to confirm that a figure exemplifying the gating strategy is provided in the Supplementary Information.
